# Recommended methods for the collection of clinical expert judgment in rare diseases: Generating evidence to support reimbursement of orphan drugs

**DOI:** 10.1017/S0266462325100457

**Published:** 2025-10-07

**Authors:** Annabel Griffiths, Lorna Dunning, Karen Facey, Dina Jankovic, Carlos González Malla, Eric Low, Michela Meregaglia, Fabian Schmidt, Kevin Wilson, Sheela Upadhyaya, Noa Chapman, Chloe Zentai, Sari Wright, Isabelle Newell

**Affiliations:** 1 Rare Diseases, Costello Medical, Cambridge, UK; 2 Medicines Evaluation Directorate, National Institute for Health and Care Excellence, London, UK; 3 Usher Institute, University of Edinburgh, Edinburgh, UK; 4 Centre for Health Economics, University of York, Heslington, York, UK; 5 University of Buenos Aires, Autónoma de Buenos Aires, Buenos Aires, Argentina; 6 Eric Low Consulting, Haddington, UK; 7 Centre for Research on Health and Social Care Management (CERGAS), SDA Bocconi School of Management, Milan, Italy; 8 Value & Access and Policy, Recordati Rare Diseases, Puteaux, France; 9 School of Mathematics, Statistics & Physics Herschel Building, Newcastle University, Newcastle, NR, UK; 10 Life Sciences Consultant, London, UK; 11 Health Policy, Costello Medical, London, UK; 12 Health Economics, Costello Medical, London, UK

**Keywords:** expert opinion, rare diseases, data collection, drug approval, clinical judgment

## Abstract

**Background:**

Developing therapies for rare diseases is challenging due to limited evidence and high degrees of uncertainty regarding the value of new treatments. Clinical expert judgment can inform modeling assumptions and address areas of uncertainty in reimbursement submissions. As current protocols do not adequately address the challenges faced in rare diseases, this research aimed to generate recommendations for the collection and reporting of clinical expert judgment in rare diseases.

**Methods:**

An international group of industry, payer, and patient experts with a background in rare diseases participated in a roundtable meeting, which aimed to identify practical challenges in and solutions for gathering clinical insights to aid reimbursement decisions for rare disease therapies. Recommendations were cocreated through iterative discussions and group agreement.

**Results:**

Developers should proactively identify uncertainties that expert judgment can address, in parallel with early evidence generation planning. Expert judgment method(s) depend on the uncertainties, with those key to decision-making requiring more robust and time-intensive methods. For highly complex and uncertain topics, methods should facilitate consensus building and expression of diverse views. Given the scarcity of rare disease experts, a high time burden falls on a few experts. Developers should engage diverse stakeholder groups to integrate broader clinical perspectives and reduce reliance on specific individuals while approaching conflicts of interest pragmatically and transparently.

**Conclusions:**

These recommendations create a blueprint for developers of rare disease therapies to conduct high-quality clinical expert judgment studies. Hence, developers can present more robust evidence to inform key areas of uncertainty in reimbursement decisions, where empirical evidence is unavailable.

## Introduction

Developing therapies for rare diseases is often challenging due to limited evidence and a high degree of uncertainty around the natural history of the condition and the value of a new treatment (1-4). Understanding disease progression under the current standard of care and how new therapies may improve outcomes is crucial to inform assessments of disease burden and treatment effectiveness. Identifying healthcare system costs associated with a condition, and robust model inputs, parameters, and assumptions, is also essential when estimating budget impact and cost-effectiveness for new treatments (1;5-10). These data are all integral to health technology assessments (HTAs) that inform reimbursement decisions.

Where clinical trial or observational data are limited, expert judgment can help address uncertainties in the evidence base (1;11). Expert judgment represents insights provided by individuals with specialized knowledge and/or experience in a particular area, which can be utilized to support quantitative analysis (e.g., by verifying assumptions and/or predicting outcomes). Although terminology can vary, expert judgment encompasses both expert opinion, which refers to qualitative information (e.g., the most common symptoms of a disease), and expert elicitation, the process through which quantitative information is obtained (e.g., estimates of the number of medical appointments an individual might have every year and how much this estimate may vary) (1;12).

Given the paucity of data regarding the natural history of rare diseases, clinical experts can provide insights into the care pathway and prognosis of specific subgroups, and in the absence of long-term data, experts can provide estimates such as expected long-term clinical outcomes for patients living with rare diseases (13;14). Various methods are available for generating clinical expert judgment, ranging from formal, structured approaches to more flexible and unstructured formats (Table 1) (1).

**Table 1.** Summary of methods available for generating clinical expert judgment during therapy development

	Method	Time burden on experts	Analysis complexity	Yields a probability distribution
Structured	SHELF (15)	Medium	High	Yes
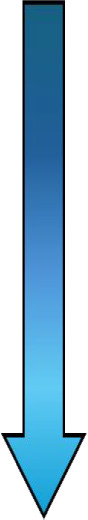	MRC Reference Protocol (16)	Medium	High	Yes
	Classical Model Method (17;18)	Medium	High	Yes
	IDEA Protocol (19)	Medium	High	No
	Delphi Method (20)*	High	Medium	No
	Surveys	Medium	Medium	No
	Expert interviews	Medium	Low	No
	Focus groups	Low	Low	No
Unstructured	Testimonies, Narratives	Low	Low	No

* The modified Delphi method is often a preferred method, as it can be conducted virtually and limits the number of prespecified survey rounds rather than continuing until 100 percent consensus is reached. This enables faster decision making and improved participant engagement while maintaining iterative feedback and expert consensus.

**IDEA, Investigate, Discuss, Estimate and Aggregate; MRC, Medical Research Council; SHELF, SHeffield ELicitation Framework.**

In recent years, HTA bodies such as the National Institute for Health and Care Excellence (NICE) and the Canada Drug Agency have stated a preference for structured expert elicitation (SEE) over other unstructured methods, in the absence of empirical data, to improve transparency and accountability in the collection and reporting of expert opinion used in decision-making (21;22). Similarly, Zorginstituut Nederland requires expert elicitation to capture uncertainty around unknown quantities or verify assumptions included in cost-effectiveness models (23).

Notably, in 2021, a reference protocol for SEE for healthcare decision-making (HCDM) was published, providing in-depth reference methods (16). The authors highlighted that there are often multiple methodological choices and that methods may need to be adapted to address practical challenges in individual settings. They highlighted limited access to experts as a common challenge encountered in rare disease settings (16). A recent evaluation of the NICE’s highly specialized technology (HST) process, which assesses and recommends specialized treatments for rare conditions, highlighted that the absence of formal guidelines on using expert judgment to support NICE’s reimbursement decisions raises concerns about the robustness of estimates derived from expert insights (24). Considering the important role of clinical judgment in rare diseases, there is an unmet need for targeted recommendations on tailoring reference protocols for rare diseases to address practical challenges commonly encountered in this context.

The recommendations outlined in this paper aim to provide a set of guiding principles for the collection of clinical expert judgment in rare diseases as a form of evidence to inform HTA decision-making and improve the robustness of estimates derived from experts. These recommendations focus on gathering expert judgment from clinical experts, such as clinicians and multidisciplinary team members, who have relevant and/or specific knowledge pertaining to a disease or therapy to generate evidence to support HTA decision-making. Clinical experts may also be invited to join appraisal committee deliberations; however, this aspect is not within the scope of this paper.

## Methods

A time-limited targeted literature review (TLR) was performed to provide a preliminary overview of the use of expert judgment in rare diseases and key stakeholders with experience in conducting or participating in such studies. Searches were conducted in PubMed on 17 February 2023 to identify studies published in the English language (Supplementary Methods). This was supplemented with grey literature searches from the International Society for Pharmacoeconomics and Outcomes Research presentations database and Google search engine. Existing literature providing guidance on how to conduct expert judgment studies was also reviewed. Records from six countries (the United Kingdom, France, Germany, Canada, Brazil, and Thailand) were prioritized, based on recommendations from authors, to capture a diverse range of HTA systems reflecting differing levels of maturity and approaches to reimbursement and cost-effectiveness assessment. This selection allowed for comparison between established bodies and those with emerging or varied methodologies, offering a broader view of global practices.

**Figure 1.**
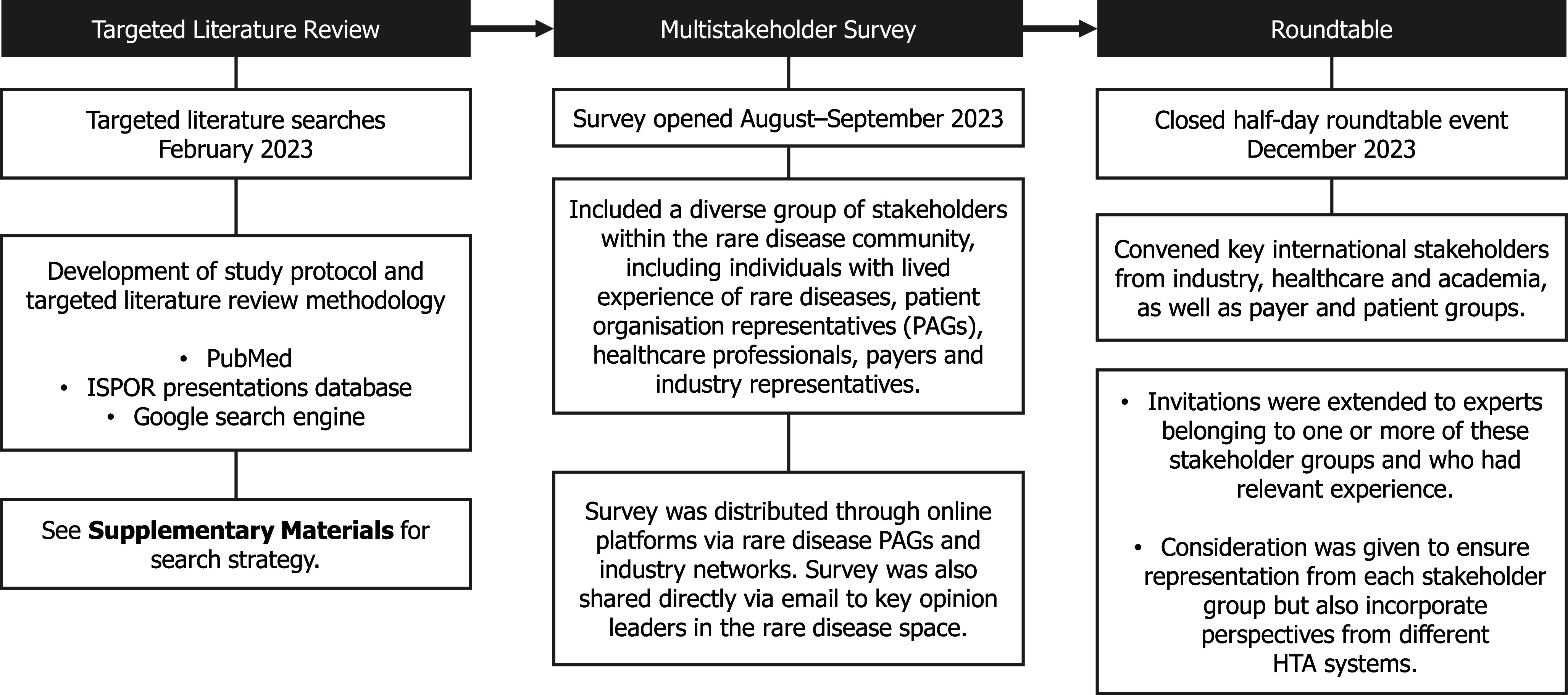
Summary of a mixed-methods approach to developing roundtable consensus recommendations. ISPOR, International Society for Pharmacoeconomics and Outcomes Research; PAG, patient advocacy group.

A global survey was subsequently conducted to understand the current use of expert judgment in developing an evidence base for rare disease therapies and identify the associated challenges (Supplementary Figure 1). The industry networks, stakeholders, and patient organizations contacted for participation in or dissemination of the survey were primarily identified from relevant records in the TLR, with further recommendations provided by the authors. The survey was also shared on LinkedIn. The challenges associated with expert judgment in rare diseases, highlighted in the survey, were used to inform discussion topics for the roundtable meeting (Figure 1). The closed half-day multistakeholder roundtable event was hosted in December 2023 in a hybrid format (i.e., both virtual and in-person) and convened thirteen experts representing industry, payer, policy, academia, and patient advocacy group perspectives (Supplementary Table 1). Invitations were extended to experts belonging to one or more of these stakeholder groups and who had relevant experience. Consideration was given to ensure representation from each stakeholder group but also to incorporate perspectives from different HTA systems. The roundtable event was separated into three sessions to identify practical recommendations for the challenges associated with the following: designing expert judgment studies; recruiting and training participants; and reporting methodology and findings. Roundtable discussions sought to achieve agreement on key challenges in each session before cocreating solutions. Recommendations were developed from these solutions by drawing on relevant literature and through iterative discussions, group agreement, and review during the development of this publication.

## Results

### Challenges

A total of 112 relevant papers were included in the literature searches, covering eighty-one rare diseases. No articles provided guidance on conducting clinical expert judgment studies in rare diseases. Identified studies predominantly sought expert opinion: seventy percent reported the collection of expert opinion, twenty-two percent discussed both expert opinion and elicitation, six percent undertook expert elicitation alone, and two percent conducted SEE. Detailed reporting of study methods was lacking among the identified publications. These findings indicate that health technology developers (HTDs) [organizations that develop new medical technologies to the standards of regulatory authorities] may face challenges in implementing and accurately reporting expert judgment methods in rare diseases.

To validate findings and identify views not reported within the literature, the survey gathered perspectives from a range of stakeholders (N = 61; Supplementary Figure 1) on the current use of expert judgment in rare diseases and the challenges associated with conducting and participating in these studies. In the survey, stakeholders were presented with a list of predefined challenges associated with conducting expert judgment studies in rare diseases and asked to select those they perceived to be the greatest challenges.

Although not all survey respondents ranked the challenges (Supplementary Figure 2 [N = 46]), these findings suggest that across all stakeholder groups, expert recruitment, logistics (including time and financial constraints), and the size of evidence gaps (areas where data and insights are unavailable) are prominent challenges for expert judgment studies in the rare disease space. These challenges were validated and discussed at the expert roundtable, where participants could also raise additional challenges.

### Recommendations

#### Study design and conduct

##### Prioritizing evidence gaps for investigation through expert judgment studies

Survey responses indicated that the extent and prioritization of evidence gaps are challenges when designing expert judgment studies in rare diseases. This is compounded by the emergence of new unforeseen gaps throughout the therapy development process.

To alleviate this, HTDs should set out an integrated evidence generation plan (iEGP) early in the therapy development process (e.g., parallel to phase II trials). The plan should identify key uncertainties and outline opportunities for expert judgment activities in the lead up to HTA decision-making. This approach reflects guidance by the study of Annemans and Makady (25), which states that rare disease HTDs should identify and list “known” and anticipated evidence gaps early in the therapy development process.

Areas of uncertainty with the greatest potential impact on decision-making should be prioritized. As part of the iEGP, HTDs should consider utilizing early economic modeling to identify key cost-effectiveness drivers. HTDs may wish to consider recruiting a small group of clinical experts (e.g., two to three individuals) to form a steering group. The steering group may provide early judgment on parameters (e.g., key assumptions, comparators, health outcomes, cost inputs) that will drive model outputs, as well as guidance throughout the development process, particularly when unforeseen evidence gaps arise.

To capture more reliable estimates, as well as the degree of uncertainty in expert responses, HTDs could utilize questions seeking upper and lower plausibility estimates only, rather than collecting individual point estimates. HTDs should also consider a mixed-methods approach to gathering expert judgment. They should prioritize areas of uncertainty, where more robust methodologies (e.g., Delphi method, SHeffield ELicitation Framework [SHELF]) may be necessary, over areas that can be addressed using unstructured methods (e.g., surveys, interviews). For example, HTDs could use SEE to elicit high-priority quantitative data, such as long-term clinical outcomes, while applying brief, unstructured methods for parameters that are less impactful on reimbursement outcomes (Box 1). This is particularly important in rare diseases, as evidence gaps are often complex and extensive and therefore require effective management of resources and expert time.

Box 1. Prioritizing evidence gaps.

**Case study:** To address uncertainties in a HTA submission and economic model for an ultra-rare primary immunological condition, a HTD conducted four targeted exercises to gather expert insights. Exercise 1 employed a modified SEE method to estimate the therapy’s long-term effects on manifestations and mortality; exercise 2 involved an EQ–5D–5L survey to collect quality of life data; and exercises 3 and 4 consisted of qualitative and quantitative surveys aimed at generating and validating key assumptions within the submission and economic model. By strategically using the modified SEE for high-priority quantitative data and assigning lower-priority data collection to surveys, the HTD effectively managed its resources, while minimizing the time required from experts (26).

##### Managing complexity and uncertainty in rare diseases

Due to limited evidence, rare disease experts are often required to provide insights into highly complex questions based on their experience, resulting in a high degree of uncertainty. Recommendations in broader guidance for expert elicitation suggest that HTDs should first conduct a pilot of elicitation protocols with a third party. Any feedback can then help refine the protocol and ensure that there is no linguistic ambiguity or framing that may lead to unwanted bias (11;19).

However, questions on complex topics may require further tailoring to help manage uncertainty. Dividing elicitation into subsections or shorter activities to allow experts to take breaks (applicable to both online and in-person studies) may allow experts to reflect on their answers. For example, while conducting an online Delphi panel, HTDs could allow experts to save their answers and return later.

To support with complexities associated with SEE, most of the current elicitation guidelines recommend providing training to participants before conducting studies. Training topics may include background on the disease, the basics of probability and uncertainty, an overview of the elicitation process, how to recognize and mitigate bias, and the study aims. Most protocols also recommend integrating a few practice questions into training materials, with some using responses to gauge experts’ skill in making judgments about uncertainty (1;11;12;16).

Training can play a key role in preparing experts to address complexities arising from extensive data gaps in rare diseases. For example, experts may benefit from training on how to express uncertainties and avoid bias (while recognizing that motivational bias is highly likely in rare diseases). Follow-up calls could be offered by HTDs to experts following training, to allow them to ask clarification questions. In addition, HTDs should provide a full and objective account of existing evidence for experts to review ahead of participation in any expert judgment study.

In the rare disease setting, expert judgment activities may involve a wide range of stakeholders from different professions/specialties (due to heterogeneity in disease manifestations) and geographies. Targeting questions to specific stakeholder groups based on their expertise minimizes the need for all clinical experts to answer every question and enables experts to answer with more certainty, while ensuring a full breadth of knowledge is captured. Consideration should also be given to the relevance (e.g., to the geography and/or healthcare system in question), language, and accessibility of questions to maximize the value of any outputs.

Where considerable uncertainties lie, consensus gathering should be prioritized, which should include an option for experts to indicate whether they feel they lack the relevant expertise to answer the question. Cross-expert interaction in “group” activities may be more valuable for gathering consensus around uncertainties in rare diseases, as opposed to activities completed by experts in isolation. This interaction will help HTDs capture a range of experiences and dissenting opinions; discussion may also help aid quicker consensus.

Weighting, where different levels of importance are assigned to quantitative values in mathematical aggregation, may be challenging in the context of expert elicitation, often due to insufficient justification for differential weights (16). However, some studies have explored performance-based weighting, which relies on responses to seed questions (1;27–31). Although current guidance states that further research on weighting methods within HCDM is needed, HTDs could explore weighting expert responses in rare indications based on the degree of specific experience, where this methodology is fully justified and transparently reported (16).

##### Managing the time burden placed on clinical experts

Survey responses indicated that for rare diseases, clinical experts are often expected to provide extensive input on many different and complex topics due to numerous evidence gaps. As such, multiple iterations and follow-up questions are often required, resulting in a significant time burden for experts, particularly where there are limited experts available.

Managing the time burden on experts appears to be largely overlooked in the existing guidance for expert elicitation. Recommendations from the broader Investigate, Discuss, Estimate and Aggregate (IDEA) protocol suggest recruiting more experts and dividing the questions between multiple groups to manage time burden, an approach that may be more feasible where numerous experts are available (19).

HTDs can take practical steps to improve the logistics of studies: for example, by carefully considering the mode and timing of delivering training (e.g., via email/separate meetings) to ensure efficient use of participants’ time in preparation for the exercise. For example, roundtable attendees recommend that a minimum of six weeks of notice is generally provided if healthcare professionals (HCPs) are required to complete prior training, with a deadline of no more than 14 days beforehand for completion; alternatively, training may be incorporated at the beginning of the session to maximize time efficiency. Transparent communication of the total expected time commitment is also essential during the invitation or contracting stage. Additionally, HTDs should ensure a streamlined contracting process. These steps will help set expectations on time commitment required, minimize the time burden on participants, and increase their willingness to engage (Box 2).

Box 2. Managing the time investment required from clinical experts.

**Case study:** Fifteen HCPs experienced in the management of people with hemophilia A in the U.K. participated in a three-round Delphi panel. Typically, each Delphi round would be conducted remotely, in the expert’s own time, with the option of a consensus meeting to facilitate cross-expert discussion and reframing of the statements. Each round may take several weeks to develop, disseminate, and receive responses on the statements. However, to reduce the time burden on participants, round 3 of this Delphi panel was conducted “live” during the in-person consensus meeting, after panelists and the steering committee had the opportunity to discuss and provide additional context for statements that had not reached consensus in round 2. This significantly reduced the time needed from experts in responding to round 3 (32).

##### Managing the emotional burden placed on experts

Roundtable participants highlighted that a high emotional burden is often placed on clinical experts who provide insights into rare diseases. In rare diseases, expert judgment can be pivotal in HCDM in the absence of clinical trial or observational data, placing significant weight on responses. As such, HTDs who conduct these studies in the rare disease space must carefully navigate the added pressure and sense of responsibility placed on rare disease experts.

Given the high responsibility placed on rare disease experts, HTDs should ensure communication is transparent, supportive, and collaborative where possible. There should be alignment at early stages on the objectives of studies and context on how expert judgment will be utilized. By also acknowledging the inherent degree of uncertainty in their insights, ensuring anonymity of granular judgments, and facilitating cross-expert deliberation, individuals may feel more supported in participating in expert judgment studies.

A summary of the recommendations on study design and conduct, as discussed by the roundtable experts, is presented in Table 2A.

**Table 2.** Recommended methods for collecting clinical expert judgment in rare diseases

Challenge	Recommendation
***A – Study design and conduct***	
There are often large numbers of extensive data gaps in rare disease evidence bases (e.g., natural history of the rare disease, disease trajectories, long-term clinical outcomes of therapies).	HTDs should proactively identify key areas of uncertainty in the evidence base at an early stage (e.g., from a reimbursement perspective, key value drivers can be determined using early health economic models). Forward planning for the collection of clinical expert judgment in rare diseases is critical and should be completed in parallel with early evidence generation planning. Where expert judgment is used to address evidence gaps, a mixed-methods approach can be applied to ensure that the most robust and time-intensive methods are appropriately prioritized for the key areas of uncertainty to effectively manage resources and expert time.
Due to limited evidence, rare disease experts are often required to provide insights into highly complex and uncertain topics. Hence, there may be a high degree of uncertainty in answers provided by an individual expert, compounded by significant variability in responses across different experts.	If the exercise involves multiple questions surrounding complex topics, HTDs should schedule regular breaks and/or provide the opportunity for experts to save their responses to return to later (if the exercise is being conducted online). Following group clinical expert judgment studies, where relevant, follow-up calls could be scheduled to provide a platform for dialogue and reflection between the experts and HTDs (e.g., allowing for discussion around particularly challenging questions where there is both uncertainty within experts and variability between experts). To address uncertainty from an individual expert, HTDs should include options for experts to indicate their uncertainty, along with the opportunity to provide a rationale (e.g., lack of relevant experience, misunderstanding the question, or limited/understudied evidence, respectively). Where possible, experts should be provided opportunities to express their uncertainty qualitatively. Where experts may not be able to respond to every question, the option for experts to answer “unknown” may be added. To manage variability of responses between experts, HTDs should encourage deliberation among experts to support the achievement of consensus. HTDs should prioritize methods that involve cross-expert interaction among differing regions and/or specialties (virtual or in-person), with support from an independent moderator for any expert interaction to enable productive discussions and manage the diverse range of participants. Where the number of experts is small and variability between experts is high, as is often the case in rare diseases, HTDs should consider exploring weighting of responses, for example based on the degree of specific experience. If applied, weighting should be fully justified and transparently reported.
A high time burden is placed on a small number of individuals due to the limited number of experts available in the rare disease space.	To minimize the time burden placed on experts, HTDs should: Evaluate the suitability of expert input for evidence gaps and assign questions to experts based on their specific experience to ensure more certain and reliable answers. Consider the type of expert providing insights and tailor the style of the question (including nuances in language, level of detail, etc.) prior to commencing the exercise, to maximize the use of experts’ time. Design and present expert judgment studies in a digestible format, with an appropriate maximum length (e.g., experts may prefer 30-minute to 1-hour sessions as opposed to multiple 20-minute segments). Transparently state during the invitation/contracting stage the expected time required from experts for the entirety of the study (including any training and briefing meetings or follow-up meetings to answer any queries, validate findings, or seek additional detail). HTDs should schedule/distribute any training sessions/materials appropriately to ensure that experts complete their training close to the exercise’s completion (e.g., no more than 14 days beforehand) and to therefore ensure experts can recall and apply any key learnings. The training should be concise and interactive to allow for any immediate questions and/or feedback. HTDs should ensure experts are paid for their time according to accepted national industry standards, ensure prompt payment, and streamline the contracting process to minimize their time burden, thereby increasing their willingness to participate.
There is a high emotional burden, pressure, and sense of responsibility placed on rare disease experts when providing their insights, as a result of the limited available evidence.	HTDs should make it clear at the invitation/contracting stage how any outputs of the exercise will be used to support rare disease therapy development (e.g., to support an industry reimbursement submission) and disseminated more widely, to ensure transparency and alignment on objectives.
***B – Expert selection***	
There is a scarcity of rare disease experts.	Given the small pool of experts in rare diseases, HTDs should look to convene mixed stakeholder groups, integrating a wider range of clinical perspectives for expert judgment studies, particularly as individuals living with rare conditions are typically managed by a wide multidisciplinary team of experts. HTDs should aim for optimal numbers where possible, as laid out in the existing protocols (e.g., 5–10 experts for SHELF or Classical method exercises). HTDs should approach COI pragmatically in the rare disease space, acknowledging that experts may advise on a range of different therapies. It is paramount that these COI are always transparently reported.
***C – Reporting and justification***	
Limited expertise and evidence in rare diseases can make existing protocols for expert judgment difficult to adhere to.	As covered by current guidance (1;10;16;36), HTDs should ensure that reporting of expert judgment studies is transparent, following accepted protocols where available. HTDs should report evidence-based justifications for where deviations from the protocol have occurred to account for nuances in the rare disease space.
The methodology and results of expert judgment studies are often not widely shared or reported beyond their intended purpose (e.g., for HCDM).	Transparently report and share the methodology of expert judgment studies, providing sufficient detail to provide accountability, to encourage best practice, and to allow for replication of studies by other members in the rare disease community. Ensure results are disseminated widely to maximize the value these findings can bring to a limited evidence base and to support activities in related rare conditions.

COI, conflict of interest; HCDM, healthcare decision making; HTD, health technology developers; SHELF, Sheffield Elicitation Framework.

#### Expert Selection

##### Ensuring a suitable and diverse range of experts

Detailed methodologies for expert recruitment were lacking in the published studies identified in the TLR. Existing guidance for expert judgment studies recommends using a transparent, systematic, and purposive sampling approach, to ensure all relevant perspectives are captured (16;19;33;34). Recruitment methods for expert elicitation studies include identifying experts through peer recognition, specialist knowledge, current work, conferences/events, and research output (11;16). Similarly, the optimal number of experts to include can vary based on time and resource constraints, with one study suggesting that the best performance is typically found with three to sixteen experts in these studies, with six being optimal for expert elicitation (35). More specifically, methods such as SHELF and the Classical method require five to ten experts, whereas larger sample sizes are achievable with other methods, such as the modified-Delphi method (16). These principles may be particularly applicable in the context of rare diseases.

Due to the inevitably limited pool of clinical experts for rare diseases, a key challenge raised in the survey and roundtable was identifying a suitable and diverse range of experts. By bringing together different perspectives (e.g., different clinical specialists, nurses, pharmacists, physiotherapists, occupational therapists), expert judgment studies can capture a wide spectrum of specific and relevant knowledge. This is particularly applicable for rare diseases with systemic symptoms affecting a variety of organs, where experience and understanding may vary drastically between experts from different medical specialties. Combining a diverse range of perspectives may help represent the heterogeneity in disease presentation and care experienced by individuals living with rare conditions. In addition, ensuring a wide scope for expert selection helps minimize selection bias by gathering perspectives across different geographical, institutional, and care levels.

Roundtable attendees noted that HTDs frequently draw from a narrow range of experts, for example often working with the same clinicians repeatedly. Due to the limited number of rare disease experts, it is likely that there will be an uneven geographical spread of experts for a specific rare disease, sometimes with multiple individuals from specialist centers. As such, HTDs may need to weigh the value of holding expert judgment studies virtually, in-person, or a combination of both. Of note, virtual activities enable additional flexibility for scheduling and could allow HTDs to gather insights from a broader geographical group. However, in-person attendance may facilitate better cross-expert deliberation, which may be beneficial depending on the chosen methodology. If taking an in-person approach, HTDs may wish to consider planning expert judgment activities around conferences where specific rare disease experts from around the world may congregate (11). This not only helps optimize attendance, but also provides experts with the opportunity to strengthen their own network.

Roundtable attendees also discussed the challenge of managing conflicts of interest (COI) among rare disease experts, given that rare disease clinical experts are often sponsored by more than one HTD or work very closely with colleagues in the industry. During expert selection, transparency about potential COI, whether motivational, intellectual, or commercial, should be documented. HTDs can include comprehensive screening questions during recruitment to identify these COI, which should be reported in any outputs. However, COI should be managed practically and not preclude participation in expert judgment studies.

A summary of the recommendations on expert selection, as discussed by the roundtable experts, is presented in Table 2B.

#### Reporting and Justification

##### Reporting and justifying methodologies

Findings from the TLR indicated considerable inconsistencies in how methods for gathering expert judgment are reported in rare diseases. These findings echo the critical review conducted by Bojke et al. (16), which observed that reporting of expert elicitation studies is generally poor, with limitations in word count for published journal articles often resulting in insufficient detail regarding reporting of SEE in healthcare contexts.

Current guidance for expert elicitation exercises strongly recommends that HTDs should systematically and transparently report their studies, as this helps improve the validity of the resulting expert judgments and will help standardize and improve elicitation applications in HTA (11;16;36). However, limited expertise and evidence in rare diseases can make existing protocols for expert judgment difficult to adhere to. Therefore, deviations from and adaptations to accepted methodological protocols should be approached transparently, with clear and systematic justification provided (e.g., with regard to expert selection, COI, and honoraria) (11;37). Transparent reporting enhances the credibility and influence of evidence derived from these studies in HCDM. As such, HTDs should ensure transparency in reporting methods and findings for all expert judgment activities, not just elicitation studies.

##### Publication and dissemination of expert judgment studies

In the literature review, it was observed that the methodology and results of expert judgment studies are often not widely shared or reported beyond their intended purpose. For example, expert judgment studies to gather evidence to support HTA are often not reported beyond this context (e.g., published as journal articles) and may not be freely available online. They can also be difficult to locate within lengthy committee papers, depending on the HTA body. Survey responses and roundtable discussions indicated that the value of reporting these studies is generally not well recognized, despite the crucial role of expert judgment in filling evidence gaps, particularly for rare diseases.

Where possible, HTDs should aim to publish findings from expert judgment studies. In doing so, other members of the rare disease community can build on previous learnings, to advance knowledge of a condition and help shape their own expert judgment studies. As HTA bodies are increasingly recognizing the value of structured expert judgment studies to inform HCDM, particularly in the rare disease space, this sharing of knowledge and experience is crucial to improving access to rare disease therapies.

A summary of the recommendations on reporting and justification, as discussed by the roundtable experts, is presented in Table 2C.

## Discussion

The recommendations provided aim to address the unmet need for tailored guidance in applying expert judgment studies to generate evidence to inform the reimbursement of rare disease therapies, drawing on relevant literature and best practice experiences shared during expert roundtable discussions. Given the complexity and nuance in the rare disease space, these guidelines should be applied pragmatically, depending on the specific context of the rare indication.

Recognizing potential COI from the involvement and remuneration of clinical experts by HTDs is crucial, as such involvement may influence perceptions by regulatory or HTA bodies, potentially restricting expert participation in processes such as submitting expert statements or participating in appraisal committee discussions. However, in smaller countries, where experts are limited, a degree of flexibility is needed to accommodate the availability of experts. By establishing clear guidelines and ensuring transparency, perceived conflicts can be mitigated, allowing all expert insights to be considered during HCDM.

Alongside clinical expert judgment, patient involvement throughout the therapy development and regulatory and HTA process is equally vital in the context of rare diseases. Methods such as the use of patient-reported outcomes (PROs), structured patient interviews, focus groups, and surveys facilitate the inclusion of patient perspectives (38–41), ensuring that both patient and clinical expertise are acknowledged within appraisal processes.

Many national HTA bodies have established supplemental appraisal processes specifically for rare disease therapies (42), integrating adapted pathways and expedited reviews and emphasizing the value of clinical and patient expert insights. With clear recommendations on the collection of clinical expert judgment in rare diseases, these guidelines provide a useful tool to better facilitate the consideration of expert insights as part of these supplemental appraisal processes, not only for HTDs but also for HTA bodies who are seeking clinical expert judgment as part of the HCDM process.

As the new European Union HTA regulation is established, offering a framework for Joint Clinical Assessment (JCA) to harmonize the evaluation of health technologies across Europe (43), it is crucial that the value of expert judgment is recognized. Notably, in the latest guidance on JCA methods and processes, there remains uncertainty around how quantitative expert judgment will be considered by JCA assessors. As more methodological guidance is published, it is hoped that the acceptability of robustly collected and clearly reported clinical expert judgment may be seen as valuable complementary evidence when empirical data are unavailable, particularly for rare diseases.

This study presents several limitations that should be acknowledged. First, the survey sample was small (N = 61), which constrains the generalizability of the findings. The challenges associated with expert judgment in rare diseases, highlighted in the survey, primarily informed the topics discussed during the roundtable. Although the sample size was small, these challenges aligned with relevant literature from the TLR and were validated by roundtable experts. Second, despite efforts to include perspectives from developed and developing countries, geographical representation remained limited; for example, no relevant records were identified for Thailand in the TLR, and there was a bias toward European respondents in the survey. Further studies could explore how considerations for expert judgment studies vary across regions in rare diseases. Third, future research could include greater representation from payers, policy makers, and clinicians, particularly from those involved in expert judgment studies in rare diseases, to further validate the recommendations.

## Conclusion

In conclusion, our recommendations provide guiding principles on how to address the unique challenges in conducting expert judgment studies in rare diseases. By taking forward these recommendations, HTDs can maximize the value of clinical expert judgment within reimbursement submissions to advance the understanding and treatment of rare diseases, ultimately improving patient access to effective treatments and therefore patient outcomes.

## Supporting information

Griffiths et al. supplementary material Griffiths et al. supplementary material

## Supplementary material

The supplementary material for this article can be found at http://doi.org/10.1017/S0266462325100457.
