# Supplementary material for: Recommended methods for the collection of clinical expert judgment in rare diseases: Generating evidence to support reimbursement of orphan drugs
Source: Int J Technol Assess Health Care. 2025 Oct 7;41(1):e76. doi: 10.1017/S0266462325100457 (PMC12592965; doi:10.1017/S0266462325100457)
Supplement: Griffiths et al. supplementary material [file S0266462325100457sup001.docx]

SUPPLEMENTARY MATERIALS

Recommended Methods for the Collection of Clinical Expert Judgment in Rare Diseases: Generating Evidence to Support Reimbursement of Orphan Drugs

***Supplementary Methods***

*Targeted Literature Review*

For the time-limited targeted literature review in PubMed, Google search engine and the International Society for Pharmacoeconomics and Outcomes Research presentations database, the search strategy was pre-defined: (rare OR orphan) AND ("expert judgement" OR "expert judgment" OR "expert opinion" OR "expert elicitation") AND (United Kingdom OR (UK OR England OR Scotland OR Wales OR “Northern Ireland”) OR France OR Germany OR Canada OR Brazil OR Thailand). Additional articles were identified based on citations within included records.

*Survey Methods*

The survey was hosted using the SurveyMonkey platform (Survey-Monkey Inc., San Mateo, California, USA; https://www.surveymonkey.com/) and was distributed to international stakeholders via email and LinkedIn (LinkedIn Corporation, Sunnyvale, California, USA; https://uk.linkedin.com/).

Supplementary Figure 1. Characteristics of participants from the multistakeholder survey*


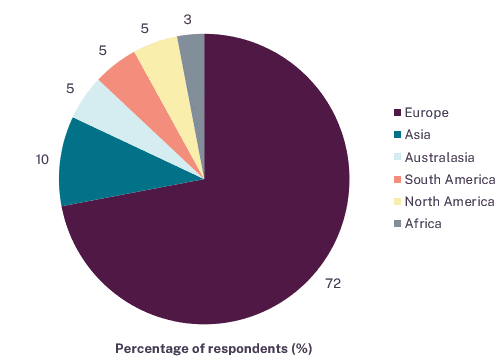

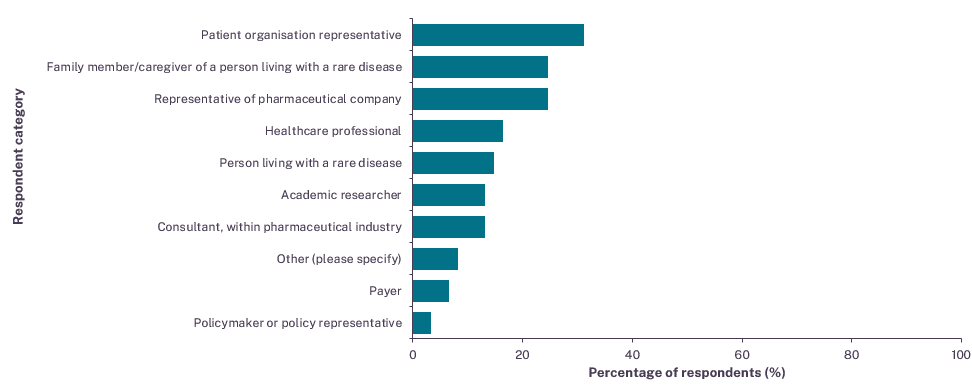

**Footnotes:** n=61 respondents. *Respondents were **not** able to select all that applied for the country they primarily live/work in however, could select all that applied for the different stakeholder groups.

Supplementary Figure 2. Challenges ranked by respondents in the multistakeholder survey*


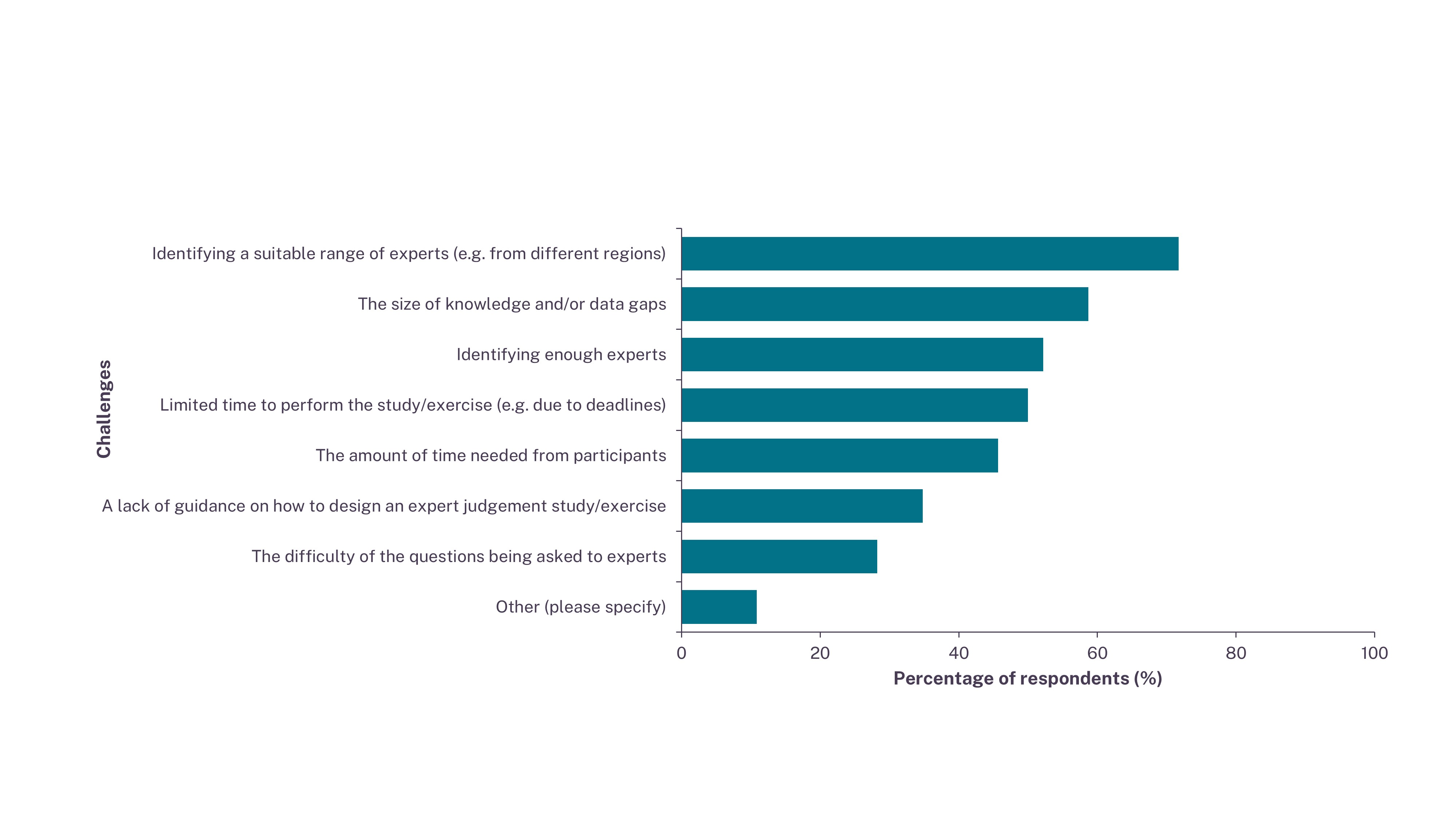
**Footnotes:** n=46 respondents. *Respondents were able to select all that applied.

Supplementary Table 1. Experts involved in the half-day multi-stakeholder roundtable event in December 2023

| **Name (A–Z)** | **Role*** |
| --- | --- |
| Annabel Griffiths | Global Head of Rare Diseases, Costello Medical |
| Carlos González Malla | Member Comisión Nacional de Evaluación de Tecnologías Sanitarias y Excelencia Clínica (CONETEC) |
| Dina Jankovic | Research Fellow, Centre for Health Economics, University of York |
| Eric Low | Steering Group, Ethical Medicines Industry Group (EMIG) |
| Fabian Schmidt | Global Head of Market Access, Health Economics and Outcomes Research (HEOR) and External Affairs, Recordati Rare Diseases |
| Isabelle Newell (Meeting Chair and Facilitator) | Consultant, Rare Diseases, Costello Medical |
| Karen Facey | Evidence Based Health Policy Consultant, with a focus on patient involvement in health technology assessment (HTA) |
| Kevin Wilson | Reader in Applied Statistics, Newcastle University |
| Lisa Foster | Director, Asia Pacific Alliance of Rare Disease Organisations (APARDO) |
| Lorna Dunning | Senior Technical Advisor, National Institute for Health and Care Excellence (NICE) |
| Lucy McKay | Chief Executive Officer, Medics for Rare Disease |
| Michela Meregaglia | Researcher, Centre for Research on Health and Social Care Management (CERGAS), Scuola di Direzione Aziendale (SDA) Bocconi School of Management |
| Sheela Upadhyaya | Life Sciences Consultant Specialising in Rare Diseases |

**Footnotes:** *The provided role/perspective reflects the experts' positions as of December 2023. **Abbreviations:** APARDO: Asia Pacific Alliance of Rare Disease Organisations; CERGAS: Centre for Research on Health and Social Care Management; CONETEC: Comisión Nacional de Evaluación de Tecnologías Sanitarias y Excelencia Clínica; EMIG: Ethical Medicines Industry Group; HEOR: Health Economics and Outcomes Research; HTA: health technology assessment; NICE: National Institute for Health and Care Excellence; SDA: Scuola di Direzione Aziendale.
